# DNA metabarcoding to unravel plant species composition in selected herbal medicines on the National List of Essential Medicines (NLEM) of Thailand

**DOI:** 10.1038/s41598-020-75305-0

**Published:** 2020-10-26

**Authors:** Santhosh Kumar J. Urumarudappa, Chayapol Tungphatthong, Pinidphon Prombutara, Suchada Sukrong

**Affiliations:** 1 Research Unit of DNA Barcoding of Thai Medicinal Plants, Department of Pharmacognosy and Pharmaceutical Botany, Faculty of Pharmaceutical Sciences, Chulalongkorn University, Bangkok, 10330 Thailand; 2 Omics Sciences and Bioinformatics Center, Faculty of Science, Chulalongkorn University, Bangkok, 10330 Thailand; 3 Microbiome Research Unit for Probiotics in Food and Cosmetics, Faculty of Science, Chulalongkorn University, Bangkok, 10330 Thailand

**Keywords:** Next-generation sequencing, Plant molecular biology

## Abstract

Traditional medicines are widely traded across the globe and have received considerable attention in the recent past, with expectations of heightened demand in the future. However, there are increasing global concerns over admixture, which can affect the quality, safety, and efficacy of herbal medicinal products. In this study, we aimed to use DNA metabarcoding to identify 39 Thai herbal products on the Thai National List of Essential Medicines (NLEM) and assess species composition and admixture. Among the products, 24 samples were in-house-prepared formulations, and 15 samples were registered formulations. In our study, DNA metabarcoding analysis using ITS2 and *rbc*L barcode regions were employed to identify herbal ingredients mentioned in the products. The nuclear region, ITS2, was able to identify herbal ingredients in the products at the genus- and family-levels in 55% and 63% of cases, respectively. The chloroplast gene, *rbc*L, enabled genus- and family-level identifications in 58% and 73% of cases, respectively. In addition, plant species were detected in larger numbers (Family identified, absolute %) in registered herbal products than in in-house-prepared formulations. The level of fidelity increases concerns about the reliability of the products. This study highlights that DNA metabarcoding is a useful analytical tool when combined with advanced chemical techniques for the identification of plant species in highly processed, multi-ingredient herbal products.

## Introduction

Traditional herbal medicines have been used in healthcare for therapeutic purposes and as dietary supplements for a very long time in many countries. The global market of herbal supplements and remedies is forecasted to grow every year and reach USD$ 117.02 billion by 2024^1^. The use of these products has gained significant momentum in the recent past, and the products are expected to have a greater demand in the near future. Accompanying this demand, however, is the danger of consuming herbal medicines and supplements that are often not reliable or unchecked. This is especially high risk in the case of traditional medicines, which are fairly far removed from modern-day science and do not undergo strict checks before entering the herbal market. Traditional herbal medicines are normally mixtures of materials from different plant species. However, in some cases, animal ingredients are also supplemented^2–4^.

In recent years, there has been increasing worldwide concern over adulteration and admixture in herbal manufacturing. Such issues can affect the growth of herbal industries and global trade by decreasing the quality, safety and efficacy of herbal medicines. These concerns are valid, as species admixture in herbal products can seriously compromise the health and well-being of consumers. Admixture can occur through a variety of processes, which may be intentional (through blatant adulteration mainly aimed at turning a profit) or unintentional (through a lack of proper quality control measures). In either case, the consequences could be adverse; hence, it is important that the products are checked, and quality assurance restored. In many cases where morphological identification of species is very difficult^3,5,6^, chemical and DNA-based approaches are used to define the biological origin of components. This is mostly applicable to traditional medicines for which the raw herbal materials are extremely processed and prepared in various dosage forms, such as capsules, tablets, and powders^2^. In Thailand, most traditional herbal products are multi-herb formulations. Despite appeals from the traditional healthcare system, there is limited scientific evidence to support the utilization of polyherbal formulations^7^. Many biological activities related to those known in the human healthcare system have been documented for polyherbal formulations. Furthermore, the use of multiple herbals or combinations of plants has been established in Indian Ayurveda^8^, Chinese traditional medicine^9^, and Thai traditional medicine^10^. Many studies have confirmed that herbal extracts from polyformulations have better efficacies than equal doses of individual active constituents and/or herbs, highlighting the significance of synergistic action in herbal medicines^11–13^. Hence, it is important to authenticate these multiple mixtures of plant samples, polyherbal formulations or herbal products to ensure their quality, safety, and efficacy^6,14^.

DNA metabarcoding has been established as an appropriate molecular technique for the authentication of plant species along with animal supplements used in different forms of traditional medicine^15–18^. Many studies have shown that herbal products/market samples are often not what they claim according to the listed plant ingredients. Using a multilocus DNA metabarcoding approach, it has been shown that only 65% of identified plant species correspond to the listed ingredients for traditional herbal products^17^. Similarly, an assessment of *Hypericum perforatum* L. samples in an herbal market revealed that only 68% of the samples were authentic^16^. Recent findings successfully demonstrated the authentication of sixteen *Veronica* herbal products that are widely used in European traditional medicine. Only 15% of the products were identified as *Veronica officinalis* L., while 62% of the products were identified as *Veronica chamaedrys* L^19^. Using next-generation sequencing, a study was performed to demonstrate the authenticity of the plant *Salvia officinalis* L. Interestingly, the additional presence of fungal species in market samples was reported^20^. Similarly, the botanical and entomological sources of honey in different commercial honey products have been identified using DNA metabarcoding regions^21^. Seventy-nine Ayurvedic herbal products were authenticated using DNA metabarcoding, and the results revealed that 67% contained a single ingredient, 21% contained multiple ingredients, and some did not contain all of the plant species mentioned on the label^15^. Much evidence supports species adulteration in herbal products, which might adversely affect consumer health and safety^6,22^. Precise and consistent sequences can be obtained for improved species determination using metabarcoding, which generates repeatable results with a reasonable cost and high-throughput sequencing^23^. Therefore, metabarcoding can be implemented for the quality control and validation of contaminants and species composition in highly processed herbal products^24^. Recent improvements in next-generation sequencing methods further reduced sequencing costs and helped improve the technique for identifying complex multi-ingredient herbal products^25^. Unless regulated, such adulteration may decrease the efficacy of herbal medicines and have adverse effects on consumer health, which will eventually result in economic losses in the herbal market^22^.

In this study, DNA metabarcoding was used to examine the plant ingredients mentioned for Thai herbal products on the NLEM from markets and hospitals. Here, we highlight metabarcoding as a potential method for use in the quality control of herbal products. Our ultimate aims are to assess the ability of DNA metabarcoding to detect the presence of plant species mentioned on the labels of herbal products and to identify the presence of any adulterants in the products.

## Results

### DNA isolation and PCR amplification

The DNA extracted from 39 samples was very inconsistent in quality and quantity. The genomic DNA concentrations of the samples were between 0.01 and 99.0 ng/μl, according to NanoDrop measurements (Table S1). The total DNA concentrations of all the samples are provided in Table S1. PCR amplification reactions were conducted for all the samples. Out of 39 samples, 33 yielded PCR amplicons from both the ITS2 and *rbc*L DNA barcode regions. The success of PCR amplification from both ITS2 and *rbc*L for the powder, tablet and capsule dosage forms was 88%, 80% and 78%, respectively. The blank (negative control) contained distilled water and yielded no molecular operational taxonomic units (MOTUs) with either ITS2 or *rbc*L primers. Six of 39 herbal samples (16%) yielded no MOTUs with the ITS2 and *rbc*L primer pairs. The failed samples (5, 9, 21, 30, 37, and 39) were not included in the results and discussion.

### Plant species detection in Thai herbal products

The labels of thirty-nine herbal products (Table 1) listed 175 medicinal plant species belonging to 136 genera and 72 families. By ITS2 metabarcoding, 1290 different plant species belonging to 138 genera and 146 families were identified, while *rbc*L analyses revealed 1,611 plant species corresponding to 138 genera and 123 families. The number of plant species detected ranged from 16 to 74 and 22 to 78 with an average of 39 and 49 species per sample when using ITS2 and *rbc*L, respectively. The raw data consisted of 5,478,664 reads, with an average of 83,010 reads per sample for both markers. After trimming and filtering, the numbers of high-quality reads were 1,989,478 and 2,552,489 with an average of 60,287 and 77,348 reads per sample for ITS2 and *rbc*L, respectively (Table S2). The total numbers of MOTUs obtained from the 33 herbal samples were 24 for the samples in powder form, 4 for the samples in tablet form, and 7 for the samples in capsule form.

**Table 1:** Details of the Thai herbal products on the National List of Essential Medicines (NLEM) used in this study.

Sample code	Herbal product name	Thai name	Sample type	Source of sample	Dosage form	Number of species mentioned in label	Number of genus mentioned in label	Number of family mentioned in label	Application/treatment
1	YA HOM THEP PA JIT	ยาหอมเทพจิตร	In-house preparation	Market	Powders	48	36	25	Dizziness relief, Cardiotonic
2	YA HOM TIP O SOT	ยาหอมทิพโอสถ	In-house preparation	Market	Powders	48	41	27	Dizziness relief
3	YA HOM IN TA JAK	ยาหอมอินทจักร์	In-house preparation	Market	Powders	49	41	30	Antiemetic, Colic relief
4	YA HOM NA VA KOT	ยาหอมนวโกฐ	In-house preparation	Market	Powders	55	46	28	Antiemetic, Antiflatulent
5	YA TAI	ยาถ่าย	In-house preparation	Market	Powders	17	14	8	Laxative
6	YA TAD BAN JOB	ยาธาตุบรรจบ	In-house preparation	Market	Powders	22	18	12	Antidiarrheal, Antiflatulent
7	YA MAN TA TAD	ยามันทธาตุ	In-house preparation	Market	Powders	27	21	14	Antiflatulent
8	YA VI SUM PA YA YAI	ยาวิสัมพยาใหญ่	In-house preparation	Market	Powders	19	15	11	Antiflatulent, Colic relief
9	YA PRA SA KAN PLU	ยาประสะกานพลู	In-house preparation	Market	Powders	30	23	17	Stomachache relief
10	YA CHAN TA LEE LA	ยาจันทน์ลีลา	In-house preparation	Market	Powders	9	8	7	Antipyretics
11	YA PRA SA CHAN DAENG	ยาประสะจันทน์แดง	In-house preparation	Market	Powders	12	12	10	Antipyretics
12	YA PRA SA MA VAENG	ยาประสะมะแว้ง	In-house preparation	Market	Powders	7	5	5	Anticough, Mucolytic
13	YA UM MA RUE KA VA TEE	ยาอำมฤควาที	In-house preparation	Market	Powders	6	5	4	Anticough, Antitussive
14	YA PRA SA JET TA PUNG KI	ยาประสะเจตพังคี	In-house preparation	Market	Powders	16	11	8	Colic relief
15	YA THO RA NEE SAN TA KAT	ยาธรณีสันฑะฆาต	In-house preparation	Market	Powders	26	23	19	Laxative
16	YA LUENG PID SA MUT	ยาเหลืองปิดสมุทร	In-house preparation	Market	Powders	13	10	10	Antidiarrheal,
17	YA MA HA JAK YAI	ยามหาจักรใหญ่	In-house preparation	Market	Powders	25	21	15	Antiflatulent, Antiemetic
18	YA KHAEW HOM	ยาเขียวหอม	In-house preparation	Market	Powders	18	17	15	Antipyretics, Anti-inflammatory
19	YA SAENG MUEK	ยาแสงหมึก	In-house preparation	Market	Powders	12	9	9	Antipyretics, Antiflatulent, Anticough, Mouth ulcer relief
20	YA TREE HOM	ยาตรีหอม	In-house preparation	Market	Powders	9	7	5	Laxative (Children), Anti-inflammatory
21	YA MA HA NIL TANG THONG	ยามหานิลแท่งทอง	In-house preparation	Market	Powders	11	11	9	Antipyretics, Anti-inflammatory
22	YA PRA SA PRAO YAI	ยาประสะเปราะใหญ่	In-house preparation	Market	Powders	21	19	12	Antipyretics, Anti-inflammatory
23	YA PRA SA KA PRAO	ยาประสะกระเพรา	In-house preparation	Market	Powders	9	6	6	Antiflatulent
24	YA PRA SA PLAI	ยาประสะไพล	In-house preparation	Market	Powders	12	7	6	Colic relief, Remedy of irregular periods
25	YA CAPSULE PRA SA PLAI	ยาแคปซูลประสะไพล	Registered herbal medicines	Hospital	Capsule	12	7	6	Colic relief, Remedy of irregular periods
26	YA PLUK FAI TAD	ยาปลูกไฟธาตุแคปซูล	Registered herbal medicines	Hospital	Capsule	8	5	5	Increase milk productions, Increase blood flow
27	YA UM MA RUE KA VA TEE	ยาอำมฤควาที ชนิดผง	Registered herbal medicines	Hospital	Powders	6	5	4	Anticough, Antitussive
28	YA PRAB CHOM PU TA VEEP	ยาปราบชมพูทวีป	Registered herbal medicines	Hospital	Capsule	4	4	4	Decongestant
29	YA PRA SA KAN PLU	ยาประสะกานพลูแคปซูล	Registered herbal medicines	Hospital	Capsule	30	23	17	Stomachache relief, Colic relief, Support digestive problem
30	YA WAN CHAK MOD LUK	ยาแคปซูลว่านชักมดลูก	Registered herbal medicines	Hospital	Capsule	4	3	3	Female fertility, Exudation of amniotic fluid, Involution of uterus
31	YA SA HAT SA THA RA	ยาสหัศธารา ชนิดแคปซูล	Registered herbal medicines	Hospital	Capsule	5	4	4	Analgesic, Anti-inflammatory, Support digestive health
32	KRA CHAI DAM	กระชายดำ	Registered herbal medicines	Hospital	Capsule	1	1	1	Dietary Supplement
33	CORDYCEPS & GINSENG	ถั่งเช่าผสมโสม	Registered herbal medicines	Hospital	Capsule	1	1	1	Dietary Supplement
34	YA BAN TAO RID SEE DUANG TA WARN	ยาบรรเทาริดสีดวงทวาร	Registered herbal medicines	Hospital	Tablets	4	4	4	Hemorrhoids relief
35	ANTIPYRETIC TABLETS (1)	ยาแก้ไข้ชนิดเม็ด	Registered herbal medicines	Hospital	Tablets	7	7	6	Antipyretics
36	ANTIPYRETIC TABLETS (2)	ยาแก้ไข้ชนิดเม็ด	Registered herbal medicines	Hospital	Tablets	7	7	6	Antipyretics
37	YA TRI PA LA	ยาตรีผลาแคปซูล	Registered herbal medicines	Hospital	Capsule	3	2	2	Mild laxative
38	INDIA SENNA CAPSULES	มะขามแขก	Registered herbal medicines	Hospital	Tablets	1	1	1	Laxative
39	YA HA RAK SA KAD	ยาห้ารากสกัดชนิดเม็ด	Registered herbal medicines	Hospital	Tablets	5	5	5	Antipyretics

**Figure 1:**
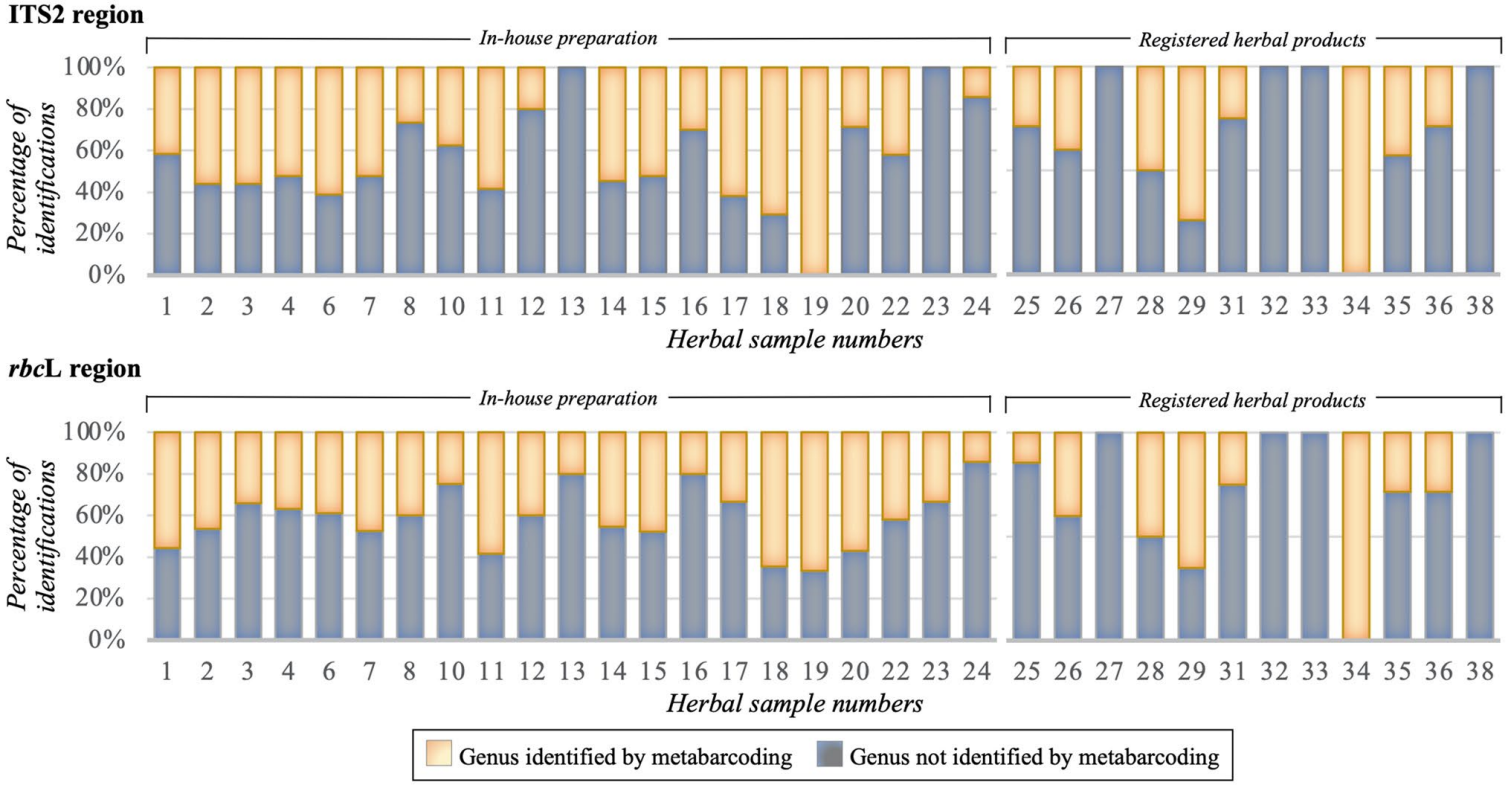
Identification of Thai herbal products at the genus level using DNA metabarcoding of the ITS2 and *rbc*L regions.

**Figure 2:**
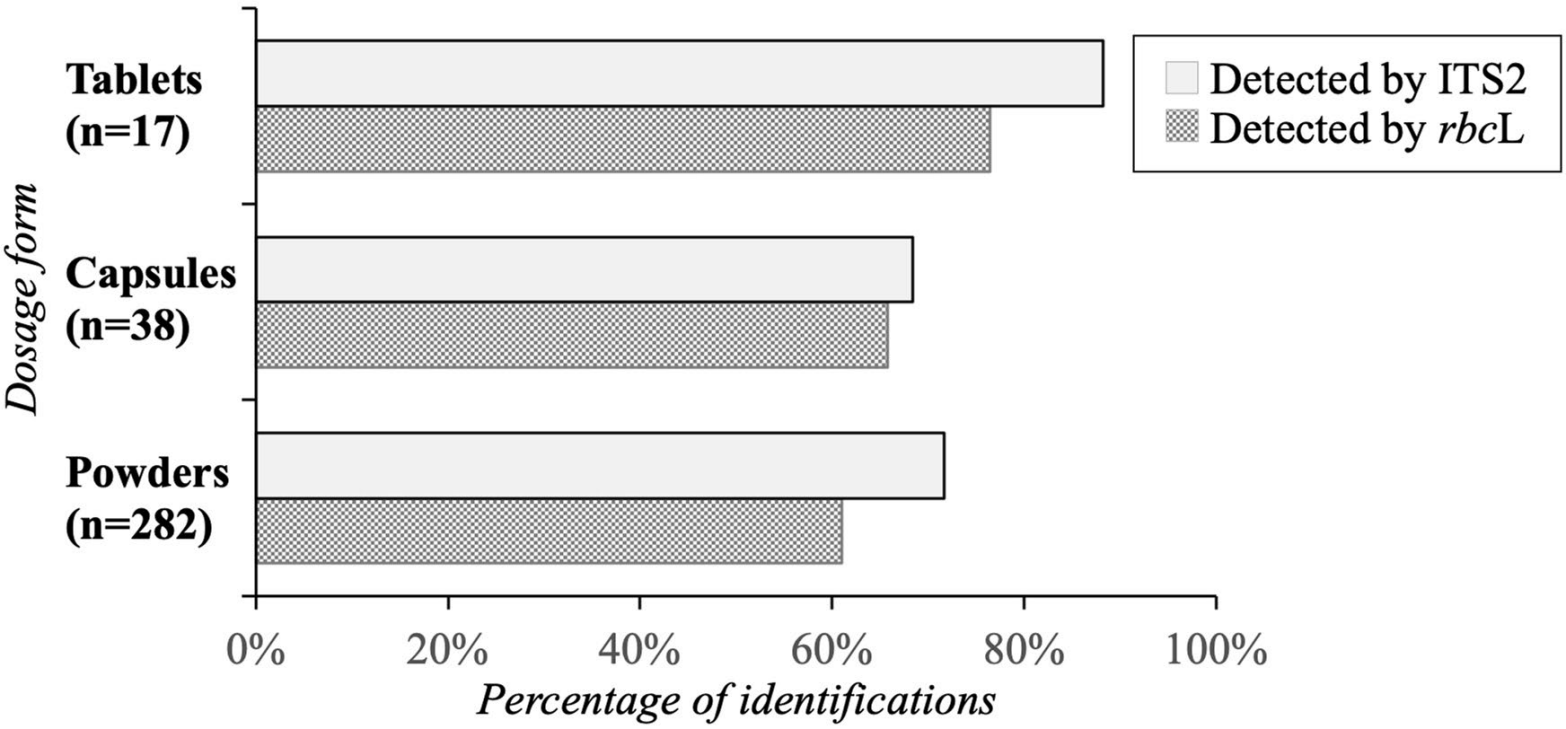
Identification of herbal products based on dosage form; ‘n’ represents the total number of plant families claimed on products’ labels.

The number of plant families detected in the herbal products ranged from 1 to 17 (Table S3). The plants listed on the labels were identified in 100% of the two single-component products and 45% of the multiple-ingredient samples (n = 31). Family-level identification of listed and non-listed (undeclared) species was performed using both ITS2 and *rbc*L (Table 2). In the ITS2 analysis, sample no. 1 in powder form contains the largest number of identified plant families (Table 2). For many samples, the families mentioned on the labels could not be identified; among those samples, sample no. 38 showed the smallest number of families that could be identified (Table 2; Fig. 1). Sample no. 2 in powder form that contained 27 families mentioned in the label, among these, 12 (45%, absolute) families were successfully identified (Table 2). Sample nos. 13, 23, 27, 32, 33, and 38 yielded 100% identifications at the genus level, and sample nos. 6, 17, 18, 29 and 34 showed the largest numbers of undetected genera among the herbal samples (Fig. 1). A separate *rbc*L analysis identified sample nos. 3 and 4, in powder form, as containing the largest numbers of identified families, with 23 and 22, respectively. Sample nos. 13, 20, 28, 31, 32, 33 and 38 contained the smallest numbers of detected families (Table 2; Fig. 1). Sample no. 1, in powder form, contained the largest number of unidentified (undetected) families, with 11 (44%, absolute), and its detected family count was 14 (56%, absolute) (Table 2). Sample nos. 13, 16, 24, 25, 27, 32, 33 and 34 yielded the largest numbers of identifications at the genus level, whereas sample nos. 18, 19, 29, and 34 showed the lowest numbers of identified genera (Fig. 1). The results from *rbc*L detection of all the herbal samples are presented in Table 2. Among the three different dosage forms, the tablet form yielded the most identifications when using both ITS2 and *rbc*L (Fig. 2).

**Table 2:** Thai herbal products and their family verification using DNA metabarcoding of the ITS2 and *rbc*L regions.

Sample number	Dosage form	Source of sample	Family listed on the label	Detected by DNA metabarcoding							
				ITS2				*rbc*L			
				Number of species	Number of family	Number of family identified (absolute %)	Number of family not identified (relative %)	Number of species	Number of family	Number of family identified (absolute %)	Number of family not identified (relative %)
1	Powders	Market	25	40	37	17 (68.00)	8 (32.00)	60	59	14 (56.00)	11 (44.00)
2	Powders	Market	27	43	40	12 (44.44)	15 (55.56)	63	62	19 (70.37)	8 (29.63)
3	Powders	Market	30	47	44	16 (53.33)	14 (46.67)	79	78	23 (76.67)	7 (23.33)
4	Powders	Market	28	40	36	16 (57.14)	12 (42.86)	79	76	22 (78.57)	6 (21.43)
6	Powders	Market	12	30	27	6 (50.00)	6 (50.00)	25	24	8 (66.67)	4 (33.33)
7	Powders	Market	14	33	29	7 (50.00)	7 (50.00)	27	24	9 (64.29)	5 (35.71)
8	Powders	Market	11	31	28	8 (72.73)	3 (27.27)	38	37	8 (72.73)	3 (27.27)
10	Powders	Market	7	52	49	6 (85.71)	1 (14.29)	33	32	6 (85.71)	1 (14.29)
11	Powders	Market	10	56	53	7 (70.00)	3 (30.00)	68	65	8 (80.00)	2 (20.00)
12	Powders	Market	5	58	53	5 (100.00)	0 (0.00)	39	38	4 (80.00)	1 (20.00)
13	Powders	Market	4	49	45	4 (100.00)	0 (0.00)	44	44	3 (75.00)	1 (25.00)
14	Powders	Market	8	49	47	6 (75.00)	2 (25.00)	47	45	7 (87.50)	1 (12.50)
15	Powders	Market	19	57	52	11 (57.89)	8 (42.11)	74	72	12 (63.16)	7 (36.84)
16	Powders	Market	10	74	68	8 (80.00)	2 (20.00)	78	77	9 (90.00)	1 (10.00)
17	Powders	Market	15	36	33	8 (53.33)	7 (46.67)	44	42	12 (80.00)	3 (20.00)
18	Powders	Market	15	65	62	7 (46.67)	8 (53.33)	65	63	7 (46.67)	8 (53.33)
19	Powders	Market	9	25	24	2 (22.22)	7 (77.78)	51	49	5 (55.56)	4 (44.44)
20	Powders	Market	5	31	29	4 (80.00)	1 (20.00)	26	25	3 (60.00)	2 (40.00)
22	Powders	Market	12	29	27	7 (58.33)	5 (41.67)	66	64	9 (75.00)	3 (25.00)
23	Powders	Market	6	41	40	6 (100.00)	0 (0.00)	50	48	5 (83.33)	1 (16.67)
24	Powders	Market	6	24	23	5 (83.33)	1 (16.67)	52	50	5 (83.33)	1 (16.67)
25	Capsule	Hospital	6	26	21	5 (83.33)	1 (16.67)	71	68	5 (83.33)	1 (16.67)
26	Capsules	Hospital	5	42	39	5 (100.00)	0 (0.00)	55	53	5 (100.00)	0 (0.00)
27	Powder	Hospital	4	33	29	4 (100.00)	0 (0.00)	34	33	4 (100.00)	0 (0.00)
28	Capsule	Hospital	4	30	29	2 (50.00)	2 (50.00)	73	71	2 (50.00)	2 (50.00)
29	Capsule	Hospital	17	38	34	8 (47.06)	9 (52.94)	53	51	9 (52.94)	8 (47.06)
31	Capsule	Hospital	4	27	24	3 (75.00)	1 (25.00)	35	33	3 (75.00)	1 (25.00)
32	Capsule	Hospital	1	40	37	1 (100.00)	0 (0.00)	31	30	1 (100.00)	0 (0.00)
33	Capsule	Hospital	1	25	25	1 (100.00)	0 (0.00)	22	20	1 (100.00)	0 (0.00)
34	Tablet	Hospital	4	16	15	0 (0.00)	4 (100.00)	34	32	2 (50.00)	2 (50.00)
35	Tablet	Hospital	6	32	31	6 (100.00)	0 (0.00)	32	30	6 (100.00)	0 (0.00)
36	Tablet	Hospital	6	37	36	6 (100.00)	0 (0.00)	28	27	6 (100.00)	0 (0.00)
38	Tablet	Hospital	1	34	30	1 (100.00)	0 (0.00)	35	34	1 (100.00)	0 (0.00)

### Plant ingredients and undeclared (undetected) taxa identified in herbal samples

**Figure 3:**
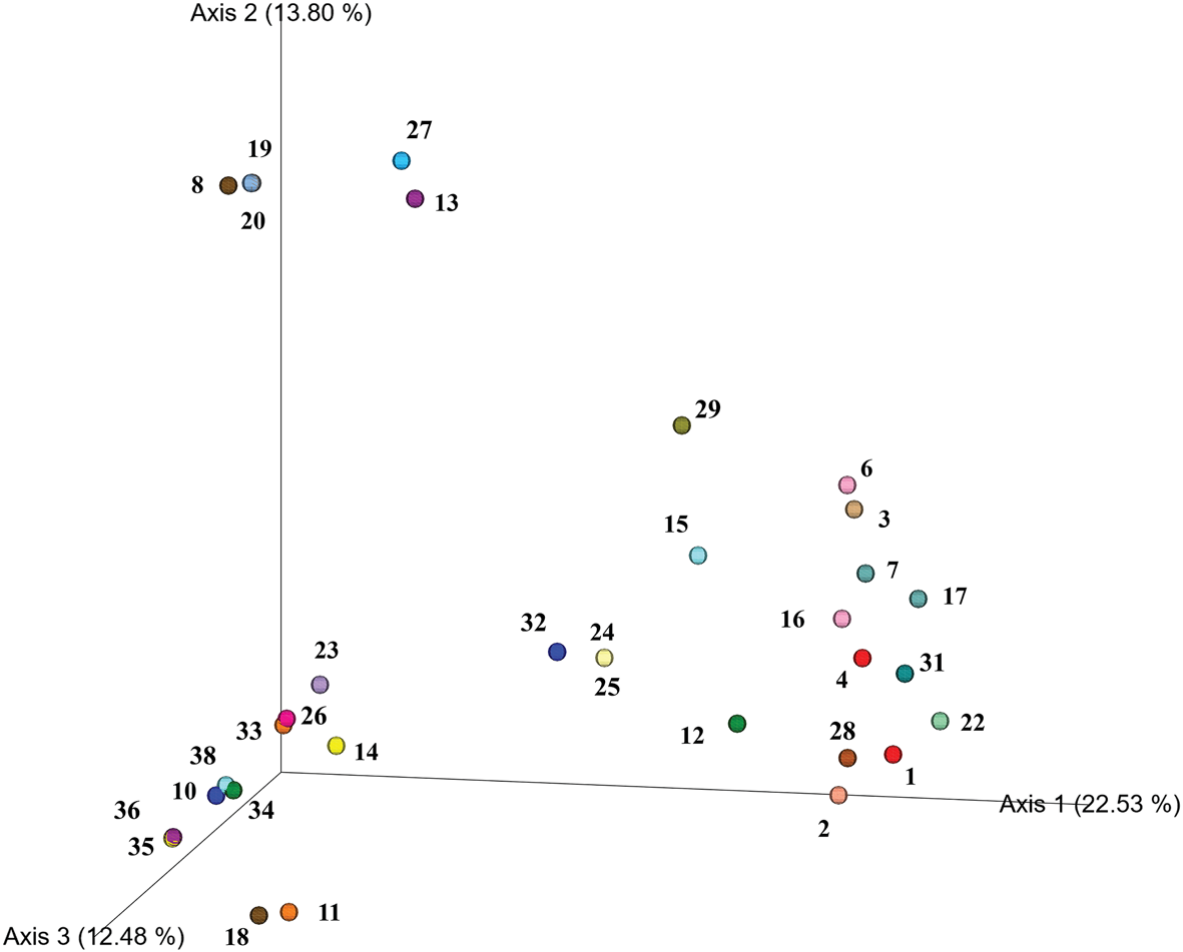
Herbal samples were grouped using principal component analysis (PCA). Each number represents a sample.

In our analysis for thirty-three herbal samples at the family level, 210 (63%) and 243 (73%) out of 337 families were identified by using ITS2 and *rbc*L, respectively (Table 2; Table S3). In separated analysis at the genus level, 242 (545%) and 256 (58%) out of 447 were identified using ITS2 region and *rbc*L region respectively (Fig. 1; Table S3). At least two to three plant species were identified in all the multiple-ingredient samples. However, among all the herbal samples, 205 and 191 of 447 species could not be identified when using ITS2 and *rbc*L, respectively (Table S4). Sample nos. 1, 2, 3, 4, 7, 8, 15, 18 and 24, which contained the largest numbers of ingredients according to the labels, yielded identifications at the family or genus level (Table 2; Fig. 1). In addition to the detected plant families, many unidentified families were observed among the herbal samples (Table S4), predominantly Apiaceae, Apocynaceae, Asteraceae, Lamiaceae, Lauraceae, Myristicaceae, Rutaceae, and Zingiberaceae (Table S4). Some identified families were not mentioned on the labels, including Araceae, Bignoniaceae, Convolvulaceae, Cucurbitaceae, Euphorbiaceae, Fabaceae, Geraniaceae, Lamiaceae, Malvaceae, Polygonaceae, Rubiaceae, Rutaceae, and Theaceae. Using a correlation matrix, principal component analysis (PCA) was performed. The plants used in the preparations of herbal samples were clustered into 3 groups. Axis 1 yielded the largest number of herbal samples clustered in groups, which were used for dizziness relief and as cardiotonic, antidiarrheal and antiflatulence agents, among other uses. Axis 2 contained fewer herbal samples clustered in groups, which were used as antipyretics and to relieve cough (Fig. 3).

### Validation of registered herbal formulations and in-house-prepared formulations

Among the thirty-nine herbal products analysed in this study, twenty-four samples were from household remedies, and fifteen samples were from registered formulations prescribed in hospitals. Our results showed that registered herbal formulations yielded the largest percentages of family-level detection, at 72% and 77% (absolute) when using ITS2 and *rbc*L metabarcoding, respectively (Table 3). Both ITS2 and *rbc*L analyses revealed that sample nos. 26, 27, 32, 33, and 38 were authentic (Table 2, Fig. 1). Similar results were obtained for all the herbal samples sample no. 34. However, genus- and family-levels detection for sample no. 34 could not be achieved with the ITS2 region, whereas *rbc*L identified two of the four families mentioned on the label. For sample no. 34, *rbc*L seems to be a good candidate region that may prove to be of greater utility. In the overall analysis, *rbc*L resulted in a higher percentage of detection in metabarcoding than the ITS2 region (Table 2, Fig. 1). Registered formulations showed larger percentages of detection at both the family- and genus-levels than household preparations when using ITS2 and *rbc*L metabarcoding. Unfortunately, we could not identify 100% authentic samples for any of the household formulations or in-house-prepared formulations using both ITS2 and *rbc*L metabarcoding. Most of the herbal samples were polyherbal formulations and highly processed powder forms. Many families that were undeclared or not identified on the label (39% and 29%, relative) were detected with metabarcoding using ITS2 and *rbc*L, respectively (Table 3).

**Table 3:** Family verification according to sample type using DNA metabarcoding.

Barcode region	Sample type	Source of sample	Mentioned in label		Detected by DNA metabarcoding			
			Number of species	Number of family	Number of species	Number of family	Family identified (absolute %)	Family not identified (relative %)
ITS2	In-house preparations	Market	463	278	910	846	168(60.43)	110(39.57)
	Registered herbal medicines	Hospital	86	59	380	350	42(71.19)	17(28.81)
*rbc*L	In-house preparations	Market	463	278	1108	1074	198(71.22)	80(28.78)
	Registered herbal medicines	Hospital	86	59	503	482	45(76.27)	14(23.73)

## Discussion

Medicinal plants and their importance in traditional healthcare systems are receiving increasing attention as solutions to resolve healthcare problems. People around the world are trying thousands of different kinds of herbal medicines each year, causing rapid growth of herbal usage and herbal industries. The quality and authenticity of herbal products directly affect the safety and efficacy of the drugs. To confirm the identity of raw ingredients or processed herbal products, quality control involving a series of standard processes and screening of labelled species along the trade chain is very important^16^. In general, herbal products are extremely processed and manufactured into capsules, tablets, powders or other forms containing many components. Although chemical and DNA-based methods can be used to identify specific targeted compounds or species of origin, they are limited in their ability to identify intrageneric substitutions and do not provide any evidence of other plant constituents in herbal samples^26^. In our DNA metabarcoding study, we used two DNA loci, ITS2 and *rbc*L, to assess the species used as ingredients in Thai herbal products. The metabarcoding method, which makes use of DNA markers with universal applicability across a wide variety of plants and animal species, enables the identification of species in herbal samples containing degraded DNA. Many reports have shown the applications of the metabarcoding technique in herbal sample validation and pharmacovigilance^4,15,16,18,27,28^ due to its cost effectiveness and ability to reveal the plant species diversity within products. However, this technique has not yet been authenticated for use in a regulatory framework for the quality control of herbal drugs^29^.

Our studies have shown that DNA barcode markers (ITS2 and *rbc*L) are useful in the identification of plant species in Thai herbal products on the NLEM. ITS2 has been revealed to be the most useful DNA marker for the identification of plants at the family level. Chen et al.^30^ demonstrated that ITS2 can serve as a universal barcode for the identification of plants at the genus- and family-levels, particularly for medicinal plants^30^, and it has been used to calculated intraspecific and interspecific distances in many studies^31^. For example, *Veronica officinalis* and *Hypericum perforatum*, which are closely related species, were successfully identified in herbal products using ITS2 metabarcoding^16^. In this study, the ITS2 and *rbc*L regions were successfully used for the identification of plants in herbal samples at the family- and genus-levels. Fungal contamination was detected in some samples, which could be due to harvesting or storage conditions^25^.

Many reports have demonstrated that the quality of extracted DNA affects PCR amplification and the success of sequencing^16,18,32^. The occurrence of DNA in processed materials is affected by degradation during harvesting, storage and manufacturing^33^. In our study, 6 of 39 herbal samples (16%) did not yield MOTUs for either the ITS2 or *rbc*L barcode. Hence, the results for these samples were excluded and not used for further analysis. Very high processing or assembly of herbs can decrease the quality of DNA, particularly if the material is extracted in hot water or liquor at high temperature. A total of 171 plant species were present in the 39 herbal products, including cultivated species (26 species), wild species (89 species) and both cultivated and wild species (56 species) (Table S5). The wild plant species detected included threatened species, such as *Enhalus acoroides* (L.f.) Royle. More of the cultivated species were herbs than were shrubs and trees. Plants in the herbal products included ferns, grasses and climbers (Table S5). In this study, sequences clustered with a 99% threshold were used to identify the best-matching species, as mentioned in published reports^16,34^. In addition to sequencing error limitations, other factors were considered to minimize errors associated with the Illumina MiSeq platform^35^, which might lead to the incorrect preparation of MOTUs. Furthermore, the strict trimming of the sequences, filtering according to nucleotide bases, quality, and length, and strict grouping criteria for MOTU formation increased our confidence in the results. In previous reports, DNA metabarcoding was used for the authentication of herbal products, and the results focused on the presence and absence of species or ingredients mentioned on the labels and whether there were any contaminants^16,18^. The presence of non-listed plant species may be due to many factors, including but not limited to unintentional adulteration and deliberate substitutions that may take place from the primary source of the medicinal plants (i.e., growing or cultivation, harvesting, storage, and transport) to the industrial processing and commercialization of herbal end-products^15^. DNA metabarcoding is an exceptionally sensitive technique, and even a small amount of DNA, e.g., contamination from pollen grains or plant residue during industrial processing, can be distinguished and recognized^15^.

According to the NLEM, many Thai herbal formulations are prepared using multiple plant species. A multitude of reports have shown that herbal formulations including single or multiple herbs have higher efficacies than formulations with single active constituents and/or herbs used alone, underlining the consequences of synergistic action in herbal medicines^11–13^. In this study, the results revealed that two herbal samples containing single ingredients were authentic. However, polyherbal samples could not be identified because most of the polyherbal formulations contained a mixture of powered samples. In ITS2 analysis, sample no. 2, in powder form, contained the most undeclared (unidentified) families (15). In a separate *rbc*L analysis, sample no. 1, in powder form, contained 14 of the families listed on the label. This could be because polyherbal powered formulations have a higher chance of admixture than other forms of samples, such as bark, leaves, stems, and roots^22^. For the polyherbal formulations, at least two to three plant species mentioned on the labels were identified. In our analyses, both regions had similar results except for sample no. 34. In general, ITS region is used extensively in herbal drug authentication including systematic and phylogenetic analysis and successfully discriminates among plant species. However, a number of concerns about the use of the ITS region for phylogenetic inference have been noted^36^. Perhaps the greatest challenge to barcoding is the presence of paralogous copies of ITS observed in some plant genera^37^; therefore, in many DNA metabarcoding studies, chloroplast regions were used along with the nuclear ribosomal ITS^15,17,18^. In sample no 34, *rbc*L seems to be a suitable region that may prove to be of great use in the identification of plant families. Among the 447 mentioned plant species, 221 and 191 could not be identified using ITS2 and *rbc*L, respectively (Table S4). Interestingly, the majority of the samples included materials from the Apiaceae, Apocynaceae, Asteraceae, Lamiaceae, Lauraceae, Myristicaceae, Rutaceae, and Zingiberaceae families (Table S4). Many families not mentioned on the label were detected, including Araceae, Bignoniaceae, Convolvulaceae, Cucurbitaceae, Euphorbiaceae, Fabaceae, Geraniaceae, Lamiaceae, Malvaceae, Polygonaceae, Rubiaceae, Rutaceae, and Theaceae. PCA was performed using a correlation matrix. The largest number of herbal samples was clustered along axis 1 (22.51%), and these herbal samples were recommended for their antidiarrheal, antiflatulence, anti-inflammation, antipyretic, and anticough effects, among others (Fig. 3, Table 1). Our results revealed that registered formulations yielded larger numbers of identifications than in-house-prepared herbal samples. Registered formulations undergo stringent quality checking by regulatory agencies before entering the market, whereas in-house formulations are prepared by local practitioners; in many cases, the latter may not pass through proper quality and safety checks. Samples in tablet and capsule dosage forms showed better results than those in powder form (Fig. 2). The samples in powder form had a higher chance of adulteration; for example, ginger powder (*Zingiber officinale* Roscoe) adulterated with chilli (*Capsicum annuum* L.)^38^ and black pepper powder (*Piper nigrum* L.) adulterated with chilli (*Capsicum annuum* L.)^39^ were reported.

The regulatory policies for herbal products vary among countries. In a few nations, such as Australia, Canada, the United States, and the European Union (EU), governing bodies/agencies evaluate the quality and safety of traditional medicines prior to permitting herbal product entry into the market^3,40^, but in rehearsal implementation, actions to control the quality and authenticity of herbal products in the marketplace seem to be limited. In several other nations, there is no well-established regulatory framework with which to evaluate the efficacy and safety of herbal drugs prior to their marketing^40^. The European Medicines Agency (EMA) regularly revises the European Pharmacopoeias^41^ and provides monographs on the quality and verification of specific herbals to provide practical methods for their quality evaluation. More recently, the British Pharmacopoeia (BP) incorporated the first universal DNA‐based identification techniques for quality control of herbal drugs, including plant sampling; DNA barcoding, isolation, and purification; PCR amplification, and a reference sequence database^40,42^. Although DNA metabarcoding is useful in the authentication of species and for analysing the species diversity in multiple mixtures of samples, it does not provide additional necessary information, such as the presence of the target compounds and their concentrations or the occurrence of chemical contaminants, which include heavy metals, irritating and disruptive dyes, and synthetic drugs^25^.

## Conclusion

Medicinal plants and their herbal products have received significant attention in healthcare systems worldwide. However, due to increasing commercial interest in using traditional medicine-based herbal products, intentional (through blatant adulteration mainly aimed at turning a profit) or unintentional (through a lack of proper quality control measures) adulteration of the plant species used in the compositions can occur. There is an urge to validate herbal products in order to gain consumer confidence by promoting and endorsing quality standards for herbal products. In this study, DNA metabarcoding provided significant testing methods for the identification of multiple mixtures of Thai polyherbal formulations/remedies. Registered herbal formulations yielded larger numbers of identifications than in-house-prepared herbal samples when the *rbc*L region was used as a marker. However, many herbal products were composed of plant species that were not listed on the labels, which should be taken into consideration by authorities to establish a requirement for herbal products in order to improve the quality control framework in the herbal market. DNA metabarcoding is not yet used as a wide-ranging authentication method in the quality control step. There is limited advocacy in relation to its importance for herbal product validation and pharmacovigilance both as a regular and a complementary technique. Hence, standardization procedures must be developed before DNA metabarcoding can be applied as a regular systematic technique and approved by expert authorities for use in a regulatory framework. A novel systematic method should ultimately use a combination of suitable chemical approaches and advanced high-throughput sequencing to analyse such mixed herbal products, concentrating on their complete trade chain, from the crude material to the processed, finalized product.

## Materials and methods

### Traditional Thai medicine (TTM) samples

In this study, 39 samples of medicines on the NLEM were collected in Thailand. Among the samples, 24 were in-house-prepared formulations (by local individuals or medicine practitioners), while 15 were formulations registered with a regulatory agency by the Thailand Food and Drug Administration (FDA) (Table S6). Herbal samples in various dosage forms, such as tablets (n = 5), capsules (n = 9) and powders (n = 25), were purchased directly from local herbal markets (n = 24) and hospitals (n = 15) in different locations in Thailand. Two groups were categorized based on label information: single and polyherbal samples. Two herbal products were composed of a single plant. For polyherbal products, the combinations of herbs included 2 to 55 plants (Table 1). In the thirty-nine Thai herbal products, 175 plant species belonging to 136 genera and 72 families were listed (Table S7). The details of the herbal products/formulations, including their Thai and English names, dosage forms, label information, and sources of collection, are provided in Table 3. The binomial names and authorities of the ingredients were validated according to The Plant List (2013)^43^.

### DNA isolation and sequencing

Genomic DNA was extracted from 100 mg of herbal products (tablets, capsules and powders) using a DNeasy Plant Mini Kit (Qiagen, Germany) according to the manufacturer's protocol and further purified using a GENECLEAN Kit (MP Biomedicals, USA) according to the developer’s instructions. The quality and quantity of the DNA were determined by a NanoDrop One UV–Vis Spectrophotometer (Thermo Scientific, USA) (Table S3) and confirmed by agarose gel electrophoresis. Purified genomic DNA was kept at − 20 °C for longer storage. Further PCR amplification with universal primers was carried out using two DNA barcoding regions, namely, the nuclear ITS2 (ITS-2F^30^ and ITS-4R^44^ primers) and chloroplast *rbc*L (*rbc*L2-F^45^ and *rbc*La-R^46^ primers) regions.

Extracted DNA from all herbal products was used for PCR in a final reaction volume of 25 µl, including 12.5 µl of template genomic DNA (20 ng/µl) and 12.5 µl of 2X sparQ HiFi PCR master mix (Quantabio, USA). The PCR procedure consisted of initial denaturation at 98 °C for 30 s, followed by 25 cycles of denaturation at 98 °C for 20 s, annealing at 55 °C for 30 s, and elongation at 72 °C for 30 s and a final elongation step at 72 °C for 5 min. Subsequently, PCR amplicons were cleaned using AMPure XP beads and indexed using 5 µl of each Nextera XT index primer in a 50 µl PCR, followed by 8–10 cycles of the PCR conditions listed above. The final PCR amplification products were purified, pooled and diluted to a final loading concentration of 6 pM. Cluster generation and 300-bp paired-end read sequencing were completed with the Illumina MiSeq platform at the Omics Sciences and Bioinformatics Center (Chulalongkorn University, Bangkok, Thailand).

### Bioinformatics pipeline

We implemented the bioinformatics pipeline described by Sickel et al.^47^ to categorize ITS2 and *rbc*L reads. In brief, raw sequence reads were obtained via the Illumina MiSeq platform and demultiplexed by MiSeq Reporter version 3.1. Forward and reverse reads were merged using the join_paired_ends.py command in QIIME v.1.8.0. Low-quality reads were filtered out with USEARCH v8.0.1477. Subsequently, high-quality reads were taxonomically categorized with the UTAX algorithm. The raw UTAX output was analysed using a custom Perl script, which tallied the number of assignments for each taxon and combined the numbers into a single table. This table was transformed into community matrix format, with columns representing samples and rows representing species, and a distinct file with the taxonomic lineage of each species was built. Species-level identifications were accepted if the queried sequences showed 99–100% matches with reference sequences and E-values below the threshold. For MOTUs based on ITS2 and *rbc*L, identifications were made to the family level and, in few cases, to the genus level because full-length *rbc*L barcodes may not provide consistency at the genus level^48^. The reference sequence database was the monthly updated NCBI/GenBank nucleotide database, and the parameters were applied to all of the samples.

### Presence and absence of species across samples

To assess the presence and absence of species in each sample, three categories were segregated based on the MOTUs from next-generation sequencing using both ITS2 and *rbc*L with the best match results. The MOTUs detected with metabarcoding in each sample were characterized as identified species (MOTUs corresponding to species listed on the sample label versus the best species match detected with metabarcoding), unidentified (undeclared) species (MOTUs corresponding to species listed on the sample label but not detected among the best matches), and other detected species (MOTUs corresponding to species not mentioned on the sample label but identified in the analysis) (Table S4). The total presences and absences of species per category (identified and not identified) were evaluated (Table S4). The counts of detected plant families and genera (identified with metabarcoding) were normalized by dividing the number of identified (identified with metabarcoding) taxa by the total number of taxa listed among the ingredients on the labels. The undeclared species (not identified with metabarcoding) were calculated by dividing the number of undeclared species (not identified with metabarcoding) by the total number of species listed among the ingredients detected with metabarcoding (Fig. 1; Table S5). We used the MOTU table as input for QIIME2 software to compute the Aitchison distance and obtained a count sum less than 10, which may represent contamination or chimeric sequences, were filtered out. Principal component analysis (PCA) was performed using the Emperor Plot functionality of QIIME2^49^. The PCA represents the beta diversity differentiating taxonomic composition between communities, and this analysis enables an overview of plant species similarity across herbal products.

## Supplementary information

Supplementary Information 1.

Supplementary Information 2.

Supplementary Information 3.

Supplementary Information 4.

Supplementary Information 5.

Supplementary Information 6.

Supplementary Information 7.

## Data Availability

The datasets generated during and/or analysed during the current study are available in the NCBI GenBank repository, SRA accession: PRJNA650007.

## References

[1] ReportBuyer. Herbal Medicine Market Size, Forecast And Trend Analysis, 2014–2024. https://www.reportbuyer.com/product/5583962/herbal-medicine-market-size-forecast-and-trend-analysis-2014-2024.html (2017). Accessed 20 Aug 2020

[2] Staats M (2016). Advances in DNA metabarcoding for food and wildlife forensic species identification. Anal. Bioanal. Chem..

[3] Coghlan ML (2015). Combined DNA, toxicological and heavy metal analyses provides an auditing toolkit to improve pharmacovigilance of traditional Chinese medicine (TCM). Sci. Rep..

[4] Cheng X (2014). Biological ingredient analysis of traditional Chinese medicine preparation based on high-throughput sequencing: the story for Liuwei Dihuang Wan. Sci. Rep..

[5] de Boer, H. J. *et al.* DNA metabarcoding of orchid-derived products reveals widespread illegal orchid trade. *Proc. Biol. Sci.* **284**, 20171182 (2017). 10.1098/rspb.2017.1182 PMC5627200 28931735

[6] Kumar SJ, Tungphatthong C, Sukrong S (2019). Mitigating the impact of admixtures in Thai herbal products. Front Pharmacol.

[7] Chanthasri W (2018). Antioxidant capacities and total phenolic contents of 20 polyherbal remedies used as tonics by folk healers in Phatthalung and Songkhla provinces Thailand. BMC Complement. Altern. Med.

[8] Kuchewar VV, Borkar MA, Nisargandha MA (2014). Evaluation of antioxidant potential of Rasayana drugs in healthy human volunteers. Ayu.

[9] Ji HJ (2016). Wuzi Yanzong pill, a Chinese polyherbal formula, alleviates testicular damage in mice induced by ionizing radiation. BMC Complement. Altern. Med..

[10] Booranasubkajorn S (2017). Vasculoprotective and vasodilatation effects of herbal formula (Sahatsatara) and piperine in spontaneously hypertensive rats. Phytomedicine.

[11] Leonard SS (2002). Antioxidant properties of fruit and vegetable juices: More to the story than ascorbic acid. Ann. Clin. Lab. Sci..

[12] Scholey AB, Kennedy DO (2002). Acute, dose-dependent cognitive effects of *Ginkgo biloba, Panax ginseng* and their combination in healthy young volunteers: differential interactions with cognitive demand. Hum. Psychopharm. Clin..

[13] Zhang A, Sun H, Wang X (2014). Potentiating therapeutic effects by enhancing synergism based on active constituents from traditional medicine. Phytother. Res..

[14] Thongkhao K, Tungphatthong C, Phadungcharoen T, Sukrong S (2020). The use of plant DNA barcoding coupled with HRM analysis to differentiate edible vegetables from poisonous plants for food safety. Food Control.

[15] Seethapathy GS, Raclariu-Manolica AC, Anmarkrud JA, Wangensteen H, de Boer HJ (2019). DNA metabarcoding authentication of ayurvedic herbal products on the European market raises concerns of quality and fidelity. Front. Plant. Sci..

[16] Raclariu AC (2017). Comparative authentication of Hypericum perforatum herbal products using DNA metabarcoding TLC and HPLC-MS. Sci. Rep..

[17] Arulandhu AJ (2017). Development and validation of a multi-locus DNA metabarcoding method to identify endangered species in complex samples. Gigascience.

[18] Ivanova NV, Kuzmina ML, Braukmann TWA, Borisenko AV, Zakharov EV (2016). Authentication of herbal supplements using next-generation sequencing. PLoS ONE.

[19] Raclariu AC (2017). Veronica officinalis product authentication using DNA metabarcoding and HPLC-MS reveals widespread adulteration with *Veronica chamaedrys*. Front. Pharmacol..

[20] Schmiderer, C., Lukas, B., Ruzicka, J. & Novak, J. J. P. m. What Else Is in *Salviae officinalis folium*? Comprehensive species identification of plant raw material by DNA metabarcoding. *Planta Med* **84**, 428–433 (2018). 10.1055/s-0043-121470 29165730

[21] Prosser SWJ, Hebert PDN (2017). Rapid identification of the botanical and entomological sources of honey using DNA metabarcoding. Food Chem..

[22] Srirama R (2017). Species adulteration in the herbal trade: causes consequences and mitigation. Drug Saf..

[23] Jia J, Xu Z, Xin T, Shi L, Song J (2017). Quality control of the traditional patent medicine Yimu Wan based on SMRT sequencing and DNA barcoding. Front. Plant Sci..

[24] Coissac E, Hollingsworth PM, Lavergne S, Taberlet P (2016). From barcodes to genomes: extending the concept of DNA barcoding. Mol. Ecol..

[25] Lo YT, Shaw PC (2019). Application of next-generation sequencing for the identification of herbal products. Biotechnol. Adv..

[26] 26Rossi Forim, M., Perlatti, B., Soares Costa, E., Facchini Magnani, R. & Donizetti de Souza, G. Concerns and considerations about the quality control of natural products using chromatographic methods. *Curr. Chromatogr.* **2**, 20–31 (2015).

[27] de Boer, H. J., Ichim, M. C. & Newmaster, S. G. DNA barcoding and pharmacovigilance of herbal medicines. *Drug Saf* **38**, 611–620 (2015). 10.1007/s40264-015-0306-8 26076652

[28] Newmaster SG, Grguric M, Shanmughanandhan D, Ramalingam S, Ragupathy S (2013). DNA barcoding detects contamination and substitution in North American herbal products. BMC Med.

[29] Agapouda A (2019). Quality control of *Hypericum perforatum* L. analytical challenges and recent progress. J. Pharm. Pharmacol.

[30] Chen SL (2010). Validation of the ITS2 region as a Novel DNA barcode for identifying medicinal plant species. PLoS ONE.

[31] Madesis P, Ganopoulos I, Anagnostis A, Tsaftaris A (2012). The application of Bar-HRM (Barcode DNA-High Resolution Melting) analysis for authenticity testing and quantitative detection of bean crops (Leguminosae) without prior DNA purification. Food Control.

[32] Raclariu AC, Heinrich M, Ichim MC, de Boer H (2018). Benefits and limitations of DNA barcoding and metabarcoding in herbal product authentication. Phytochem. Anal.

[33] Novak J, Grausgruber-Groger S, Lukas B (2007). DNA-based authentication of plant extracts. Food. Res. Int..

[34] Veldman S (2017). High-throughput sequencing of African chikanda cake highlights conservation challenges in orchids. Biodivers Conserv.

[35] Schirmer M (2015). Insight into biases and sequencing errors for amplicon sequencing with the Illumina MiSeq platform. Nucleic Acids Res.

[36] Álvarez I, Wendel JF (2003). Ribosomal ITS sequences and plant phylogenetic inference. Mol. Phylogenetics Evol.

[37] Campbell CS (2005). Nuclear ribosomal DNA internal transcribed spacer 1 (ITS1) in Picea (Pinaceae): sequence divergence and structure. Mol. Phylogenetics Evol.

[38] Dhanya K, Sasikumar B (2010). Molecular marker based adulteration detection in traded food and agricultural commodities of plant origin with special reference to spices. Curr. Trends Biotechnol. Pharm..

[39] Parvathy VA (2014). DNA barcoding to detect chilli adulteration in traded black pepper powder. Food Biotechnol..

[40] Sgamma T (2017). DNA barcoding for industrial quality assurance. Planta Med.

[41] European Pharmacopoeia, 8th Edition. Council of Europe: Strasbourg. *EDQM* (2014).

[42] Heinrich M, Anagnostou S (2017). From pharmacognosia to DNA-based medicinal plant authentication: pharmacognosy through the centuries. Planta Med..

[43] The Plant List. Version 1.1. (2013).

[44] White, T. *et al.* PCR protocols: a guide to methods and applications. (1990).

[45] Palmieri L, Bozza E, Giongo L (2009). Soft fruit traceability in food matrices using real-time PCR. Nutrients.

[46] Kress WJ, Erickson DL (2007). A two-locus global DNA barcode for land plants: the coding *rbc*L gene complements the non-coding trnH-psbA spacer region. PLoS ONE.

[47] Sickel W (2015). Increased efficiency in identifying mixed pollen samples by meta-barcoding with a dual-indexing approach. BMC Ecol.

[48] Hawkins J (2015). Using DNA metabarcoding to identify the floral composition of honey: a new tool for investigating honey bee foraging preferences. PLoS ONE.

[49] Martino C (2019). A novel sparse compositional technique reveals microbial perturbations. MSystems.

